# Extracardiac Compression by Gastrointestinal Structures: A Comprehensive Anthology From the Literature

**DOI:** 10.1155/crp/5871029

**Published:** 2025-04-23

**Authors:** Riccardo Scagliola, Rosario Fornaro, Sara Seitun

**Affiliations:** ^1^Cardiology Division, Cardinal G. Massaia Hospital, Asti, Italy; ^2^Department of Surgical Sciences and Integrated Diagnostics, University of Genoa, Genoa, Italy; ^3^Radiology Division, San Martino Hospital, Genoa, Italy

**Keywords:** clinical findings, ECG changes, extracardiac compression, gastrointestinal structures, imaging tools

## Abstract

Extrinsic heart compression by gastrointestinal (GI) structures is an often underrecognized finding in clinical practice. It is potentially related to unpredictable clinical conditions, ranging from incidental detection in asymptomatic subjects, to deranging and potentially life-threatening clinical manifestations. However, despite its potential clinical relevance, there is still no comprehensive analysis investigating the surrounding causes, clinical findings, and diagnostic imaging work-up for this patient population. A narrative review with an extensive bibliographic search of the literature was performed using PubMed (MEDLINE), Embase, and Cochrane Central Databases up to December 31, 2023. Despite the broad spectrum of GI etiologies, clinical manifestations, and cardiac chamber involvement scenarios, physicians must be aware of such an uncommon condition, in order to provide timely diagnosis through a comprehensive imaging approach, avoid misleading interpretations, and determine the most appropriate decision-making strategy.

## 1. Introduction

Extrinsic heart compression by mediastinal structures is seldom reported in the literature, with gastrointestinal (GI) etiologies being the most frequently reported causes of extracardiac impingement [1, 2]. Albeit uncommon, pathologic interactions between GI structures and cardiac chambers may often lead to unpredictable diagnostic pitfalls in clinical practice because of their close proximity. Time of presentation and clinical manifestations are widely heterogeneous, ranging from incidental detection in asymptomatic subjects to deranging and potentially life-threatening clinical conditions. Despite sporadic reports describing its clinical impact on the general population, there is still no comprehensive analysis investigating the surrounding causes, demographics, clinical findings, and diagnostic workup of extrinsic heart compressions by GI structures. This narrative review aims to provide a comprehensive overview focusing on etiopathogenesis, anatomical findings, clinical manifestations, and diagnostic imaging workup of this uncommon condition, in order to improve knowledge for a proper decision-making approach and raise awareness of its potential implications in clinical practice.

## 2. Methods

An extensive bibliographic search of the literature was performed, including PubMed (MEDLINE), Embase, and Cochrane Central Databases, from January 1, 1984, to December 31, 2023. The following keywords were searched (in the title and/or abstract): (“cardiac” OR “heart” OR “atrial” OR “ventricular”) AND (“compression” OR “impingement” OR “proximity” OR “encroachment” OR “impression” OR “displacement”). Two reviewers (R.F. and S.S.) independently and separately collected data using a standardized data abstraction form. Any discrepancies were resolved by discussion and consensus. All available case reports and case series written in English, reporting information on epidemiology, etiopathogenesis, clinical findings, and diagnostic imaging workup of subjects with extracardiac compressions by GI structures, were included in our search material.

## 3. Results

Out of 6021 papers initially retrieved, 1275 duplicates and 684 records in languages other than English were removed. Following a thorough screening of the literature search, of the 4062 remaining records 3574 were ruled out as they did not pertain to the research topic. Additionally, 363 records were ruled out because they were one or more of the following: records of subjects aged < 18 years; papers without full-text available; and papers without individual case data. The remaining 125 full-text records were included in the search material.

## 4. Discussion

### 4.1. GI Etiologies

#### 4.1.1. Hiatal Hernia (HH)

**Figure 1:**
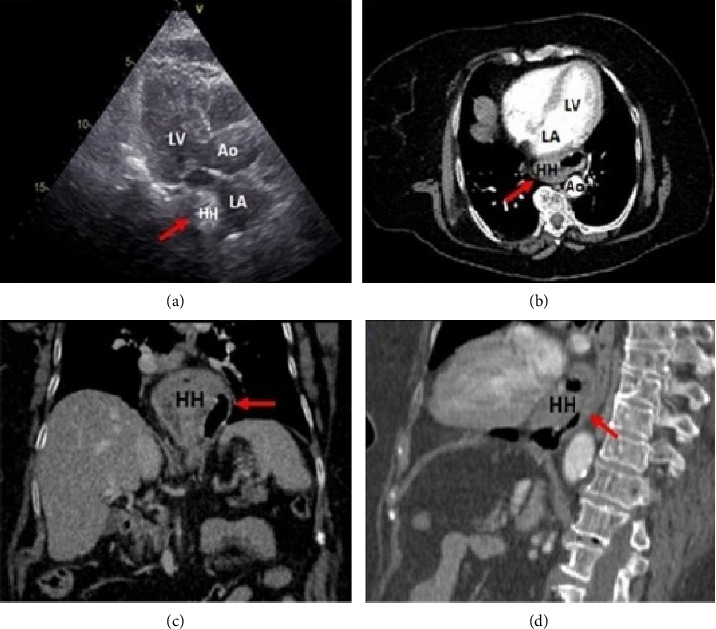
(a) Transthoracic echocardiography, parasternal long-axis view showing an echolucent extrinsic mass compressing the posterior aspect of the left atrial wall (red arrow). (b–d) Thoracoabdominal computed tomography showing extrinsic left atrial compression by a large hiatal hernia, respectively, at the transverse, coronal, and sagittal sections through the left atrium (red arrow). Abbreviations: Ao: aorta; HH: hiatal hernia; LA: left atrium; LV: left ventricle.

HH accounts for most extrinsic cardiac compressions with more than half of the total cases reported in the literature (Figure 1). Specifically, giant HH—containing more than 30% of the stomach intrathoracically—is more liable to develop symptomatic extrinsic cardiac impingement. This is usually exacerbated by the forward-leaning position, especially after large meals [3, 4]. Both sliding and paraesophageal HH have been potentially related to extrinsic cardiac impairment. In the former, accounting for over 90% of total cases, the gastroesophageal junction is usually displaced > 2 cm above the esophageal hiatus, whereas in paraesophageal herniations the gastroesophageal junction remains in its normal position, and a portion of the stomach herniates above the diaphragm. Two main pathogenic mechanisms are posited to play a pivotal role in developing enlarging HH: gastroesophageal reflux disease, which leads to the scarring and shortening of the esophagus, thus causing traction of the gastroesophageal junction and gastric herniation, and chronic positive pressure of the esophageal diaphragmatic hiatus, which predisposes to gastric displacement into the chest [5, 6]. Albeit seldom reported, large HHs may lead to gastric volvulus, which occurs when the stomach rotates by more than 180°, either along its longitudinal (“mesenteroaxial”) or transverse (“organoaxial”) visceral axis. In this clinical scenario, the resulting distention of the thoracic portion of the stomach may be enough to worsen the compression of mediastinal structures—including the left-side heart chambers—leading to gastric necrosis and rupture due to impaired wall perfusion [7].

#### 4.1.2. Achalasia

**Figure 2:**
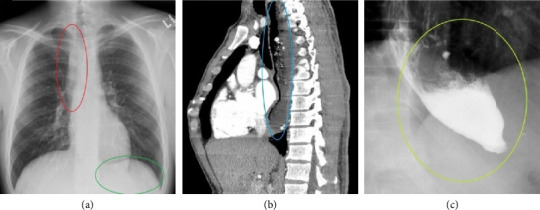
(a) Chest X-ray showing a convex opacity overlapping the right mediastinum (red oval) and the absence of a gastric bubble (green oval). (b) Chest computed tomography, sagittal section through the left atrium showing tracheal and left atrial compression from an enlarged esophagus (blue oval). (c) Barium swallowing radiogram showing a “bird's beak” or “rat's tail” appearance of the lower esophagus (yellow circle) with no contrast trickling into the stomach (adapted from Bhamrah et al.) [10].

In other circumstances, extracardiac impingement is caused by an abnormal distension of organs in their normal position rather than a displaced GI structure. This is the case of achalasia, a functional esophageal motor disorder representing the second most prevalent GI cause of extracardiac compression. In this clinical condition, the pathogenic mechanism of extrinsic impairment involves a failure in organized esophageal peristalsis caused by a degeneration of the inhibitory neurons of the Auerbach/myenteric plexus. This in turn leads to impaired relaxation of the lower esophageal sphincter, resulting in increased basal sphincter pressure, food stasis, and distension of the distal esophagus with no visceral displacement [8, 9]. Several radiographic findings may help in diagnosing achalasia, including a small or absent gastric bubble; the presence of air-fluid levels in the thoracic esophagus due to stasis with retention of food and secretion; and anterior displacement of the trachea in the lateral view. Additional radiological findings by barium swallowing study include a tapering of the lower esophagus (generally referred to as “*bird's beak*” or “*rat's tail*”) with no contrast seepage into the stomach and upper esophageal dilatation with food debris [10] (Figure 2). When achalasia is clinically and radiologically suspected, esophageal manometry is mandatory for diagnosis confirmation, as it can detect abnormal esophageal peristalsis and the lack of lower esophageal sphincter relaxation [11].

#### 4.1.3. Esophageal Tumors

**Figure 3:**
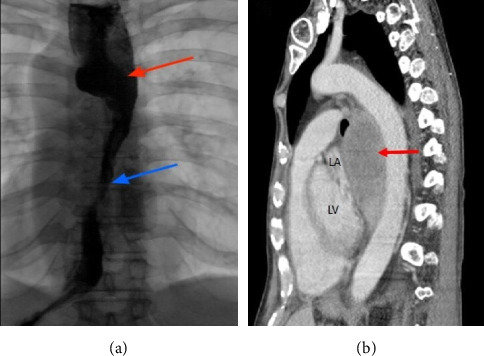
(a) Barium swallowing radiogram showing proximal esophageal dilation (red arrow) and midesophageal narrowing (blue arrow). (b) Chest computed tomography, sagittal section through the left atrium showing significant left atrial compression from an esophageal mass (red arrow) (adapted from Polina et al.) [12]. Abbreviations: LA: left atrium; LV: left ventricle.

Benign or malignant forms of esophageal tumors—either primitive or metastatic neoplasms—can potentially be involved in extrinsic heart impingement and anatomical distortion of the cardiac chambers. This is especially true for exophytic lesions growing out of the esophageal surface, which may predispose to extracardiac impairment because of their extrinsic mass effect [12] (Figure 3). In this clinical context, Bayraktar et al. reported the case of a patient with atrial fibrillation triggered by esophageal squamous cell carcinoma compressing the LA [13]. In the same way, Cakar and colleagues described a case of extrinsic LA compression by a leiomyoma of the distal esophagus, treated with surgical enucleation [14].

#### 4.1.4. Iatrogenic Causes

**Figure 4:**
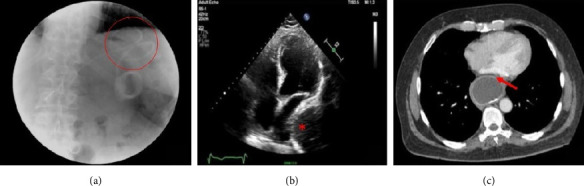
(a) Barium swallowing radiogram showing laparoscopic adjustable gastric banding rotation resulting in band slippage (red circle). (b) Transthoracic echocardiography, apical 4-chamber view showing left atrial compression from megaesophagus during diastole (red asterisk). (c) Computed tomography, transverse section showing megaesophagus causing extrinsic left atrial compression (red arrow) (adapted from De Silva et al.) [19].

**Figure 5:**
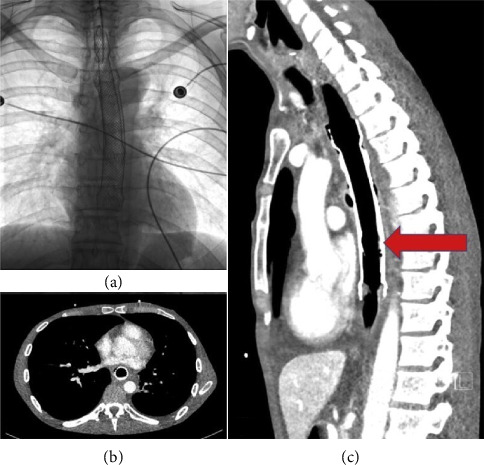
(a) Fluoroscopy showing the radiologic appearance of a stent-in-stent at the time of esophageal stent implantation. (b and c) Computed tomography, transverse, and sagittal sections showing partial compression of the posterior left atrial wall by an esophageal stent. Note the termination of the inner stent at left atrium level (red arrow) (adapted from Mazzella et al.) [20].

Interventional procedures are responsible for a broad spectrum of potential causes of extrinsic cardiac impairment. Megaesophagus has been detected in 1.9% of patients after laparoscopic adjustable gastric banding and has been arbitrarily defined as a change in esophageal width greater than 30% as compared to baseline, or > 35 mm regardless of perioperative esophageal size (Figure 4) [15–18]. Predisposing conditions for developing megaesophagus in this postoperative setting include poor compliance with diet in physiologically unprepared patients, as well as band slippage or overinflation; this can impair relaxation of the lower esophageal sphincter, leading to esophageal dilatation and extrinsic heart impairment [19]. Additionally, iatrogenic causes of extracardiac impingements have also been noticed following reconstructive esophageal surgery for neoplastic conditions or as a consequence of stent placement, either to palliate advanced malignancies or to treat nonneoplastic lesions (e.g., benign strictures, spontaneous perforations, caustic injuries, postoperative anastomotic leakages, inflammatory, or congenital esophageal fistulae) [20–23] (Figure 5).

#### 4.1.5. Congenital and Traumatic Diaphragmatic Herniations

Other conditions leading to extracardiac compression include congenital and traumatic diaphragmatic herniations, resulting in the abdominal viscera protruding into the chest cavity due to compromised diaphragmatic integrity. Congenital diaphragmatic herniations are related to embryologic developmental defects of the diaphragm, leading to the herniation of abdominal viscera content into the thorax. They are the most prevalent GI cause of extracardiac impingement in children, with an incidence of 0.8–5/10,000 births. Few cases are reported in adults, most frequently when diaphragmatic developmental defects remain undetected in childhood, resulting in the herniation of abdominal viscera into the chest [24, 25]. Among them, *Bochdalek hernias* are the most common form of congenital diaphragmatic herniations, accounting for more than 90% of total cases. They most frequently occur in the posterolateral left side of the diaphragm compared to the right side (85% vs. 15% respectively). The condition becomes symptomatic when pulmonary hypoplasia occurs, as the presence of abdominal viscera in the chest cavity during the prenatal period often prevents or delays lung development [26, 27]. By contrast, *Morgagni–Larrey hernias* account for less than 5% of total congenital diaphragmatic herniations. They are caused by anterior diaphragmatic defects developing from a partial failure in muscle fiber migration covering the foramen of Morgagni (the space located between the sternum and the costal borders). Because of their retrosternal or parasternal location, they are often incidentally detected in totally asymptomatic subjects [28]. Extracardiac compression may also be caused by diaphragmatic herniations following traumatic injuries. This condition occurs in approximately 0.8%–3.6% of thoracoabdominal traumas, in relation to either blunt or penetrating injuries. More than 90% of acquired diaphragmatic herniations follow blunt traumas, while diaphragmatic tears from penetrating injuries are seldom reported [29–31]. In this clinical context, the diaphragmatic herniation of abdominal viscera is favored by a sudden increase in the pleuroperitoneal pressure gradient triggered by the trauma. This overcomes the tensile strength of the diaphragm, especially in potentially weak areas along the embryological fusion points, which are more vulnerable to increases in intra-abdominal pressure [32].

#### 4.1.6. Other Etiologies

Other predisposing conditions for extracardiac impingement have been anecdotally reported (Table 1). Cases of extrinsic LA compression by an esophageal bolus have been noticed, particularly in the case of impaired peristalsis related to either structural or neuromuscular disorders [33, 34]. Furthermore, esophageal dissecting hematomas have also been described as a liable cause of LA collapse and hemodynamic impairment. Their pathophysiology is characterized by submucosal hemorrhage occurring at the distal esophagus due to reduced support by adjacent structures. Potential etiologies include a sudden increase in pressure in the esophagus (e.g., during swallowing or vomiting), possibly associated with a temporary loss of coordination in the opening mechanisms of the upper and lower esophageal sphincters, and endoscopic procedures, bleeding disorders, or predisposing pharmacological therapies (e.g., patients under treatment with antiplatelets, anticoagulants, or thrombolytics) [35, 36]. Finally, extracardiac compression has also been reported following visceral angioedema. In this clinical context, the sudden accumulation of interstitial fluid and vasodilatation of the mucus membrane in the esophagus results in esophageal swelling, which in turn may lead to extrinsic cardiac impairment [37].

### 4.2. Location

**Figure 6:**
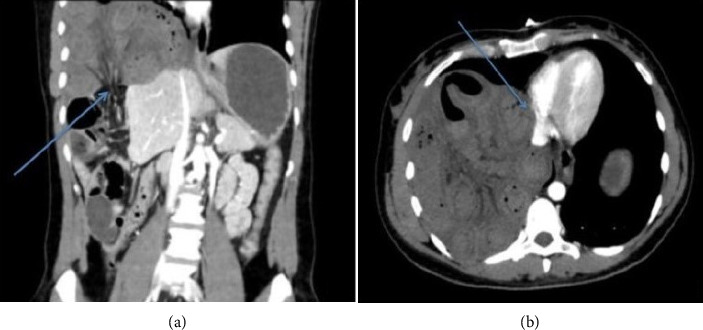
(a) Computed tomography, coronal section showing right diaphragmatic herniation (blue arrow). (b) Computed tomography, transverse section showing digestive handles in the right thorax cavity compressing the right-side heart chambers (blue arrow) (adapted from Deniau and Beloucif) [42].

With regard to potential cardiac impairment by adjacent mediastinal structures, the left-side heart chambers are more frequently affected by extrinsic GI compression compared to the right-side chambers, mainly due to the posterior mediastinal location of gastroesophageal organs. Specifically, the LA is most often affected by extrinsic compression due to its topographic and hemodynamic features, particularly its inferior and posterior cardiac location, thin wall, and low intraluminal pressure, making the LA prone to extrinsic impressions by neighboring structures [3, 38]. Based on the presence of anatomical deformation and hemodynamic compromise, D'Cruz et al. distinguished between two types of patients: those with LA “obstruction,” where atrial displacement resulted in symptoms of hemodynamic impairment caused by significant mitral inflow obstruction, and those with LA “encroachment” and “proximity,” referring, respectively, to asymptomatic subjects with or without cardiac chamber deformation, in whom extrinsic compression was incidentally detected by imaging tools [1]. Furthermore, Naoum et al. proposed a further classification of LA compression based on the LA contour as seen in two orthogonal views in computed tomography (CT) scans. A convex LA appearance in both views was considered normal, while LA compression was defined as “mild” when the LA contour was flattened in one view; “moderate” when flattened in both views; and “severe” in the case of a concave LA shape in either view [39]. Extrinsic impingement of the left ventricle (LV) by adjacent GI organs has also been described, albeit with a lower prevalence compared to LA mainly due to LV's higher intracavitary pressure. In this setting, extrinsic LV compression may induce transitory midventricular or LV outflow tract obstruction or compromise the mitral transvalvular flow at the LV inflow tract [40, 41]. Finally, gastroesophageal causes are responsible for right-side heart compression only in a minority of subjects, as the anterior location of the right cardiac chambers does not predispose to anatomical compromise by posterior mediastinal structures. In this regard, right-sided heart impingement has occasionally been reported in subjects with Morgagni–Larrey herniations, despite its low incidence in the general population [28, 29, 42] (Figure 6). More frequently, right-sided cardiac compression is notable following anterior mediastinal reconstructive esophageal surgery. This is because the close proximity of the reconstructed retrosternal digestive tract to the right cardiac chambers overthrows the physiological anatomical relationship between the esophagus and the heart [43, 44]. Right-side diaphragmatic tears and traumatic herniations are also extremely rare due to the right hemidiaphragm being protected by the large size of the liver beneath it. When they do occur, they are generally associated with higher morbidity and mortality rates [30, 31].

### 4.3. Clinical Presentation

Most subjects with extracardiac impairment by GI structures are symptomatic at the time of diagnosis. Dyspnea and exercise intolerance are the most commonly reported complaints, accounting for nearly 50% of total cases, with a significantly higher prevalence among subjects with left-side cardiac compression. Several causes are postulated to produce dyspnea in this kind of patient, either in relation to displaced GI organs (as in the case of enlarging HH) or abnormally distended organs in their normal position (as for achalasia) [45]. This frequently results in the enlarging structure producing a significant mass effect into the chest, causing LA compression and consequently dyspnea and/or exercise intolerance. This is either due to a decreased stroke volume and cardiac output as a consequence of reduced LV preload or to pulmonary venous congestion due to increased LA filling pressure and backward transmission to pulmonary circulation [1, 46]. In this clinical scenario, dyspnea is usually exacerbated by lying down, typically after large meals, which increases the distension of the extrinsic GI structure and worsens extracardiac impairment. Anginal-like chest pain is also a commonly reported finding at clinical onset. Proposed causes of chest pain in this clinical setting include (i) a mismatch between oxygen supply and demand related to impaired stroke volume and reduced cardiac output; (ii) microvascular disruption with changes in coronary reserve flow; (iii) direct compression and geometrical disarrangement of epicardial coronary vessels by extrinsic structures [40, 47–49]. Additionally, syncopal episodes are also reported as a clinical manifestation of extrinsic heart impingement in this population subset, especially when triggered by swallowing, heavy meals, and recumbency and exacerbated by forward-leaning positions [48]. The pathogenesis of syncope in this clinical setting is thought to be driven by two possible mechanisms. One involves the shared cardiac and esophageal innervation through the vagus nerve, resulting in cardioinhibitory or vasodepressor responses. Esophageal wall stretching activates mechanoreceptors, which send signals to the brainstem along the esophageal plexus. Efferent impulses from the brainstem reach the sinoatrial and atrioventricular nodes through the right and left vagus nerves, respectively, leading to several types of paroxysmal bradyarrhythmias such as sinus bradycardia, sinus arrest, or advanced atrioventricular blocks. Increased vagal tone may also induce syncope through sympathetic withdrawal, leading to systemic vasodilatation and hypotension [10, 50, 51]. The second pathogenic mechanism involves transitory mechanical compression of the LA by lying intrathoracic stomach or dilated esophageal segments, resulting in paroxysmal mitral flow obstruction, decreased cardiac output, and transient cerebral hypoperfusion [1, 52]. Diagnostic findings suggesting a suspected extrinsic GI-related etiology of syncopal episodes include a temporary correlation between loss of consciousness and potential triggers, like the postprandial period; forward-leaning positions increasing the severity of LA compression by the adjacent intrathoracic GI structures; and responsiveness after interventional or pharmacological treatment of the underlying GI cause [53, 54]. Albeit less frequently reported, coughing shares the same pathogenic mechanism as syncopal episodes in this kind of patient, involving direct mechanical compression of the afferent vagal fibers by enlarged gastroesophageal viscera (as they both pass through the diaphragmatic hiatus) and the transmission of signals to the central nervous system. However, coughing may also be related to other clinical conditions like recurrent gastric acid reflux and esophageal tissue erosion, which are commonly reported in gastroesophageal disorders, regardless of extrinsic cardiovascular displacement [55, 56]. Finally, extrinsic heart impingement may be also incidentally detected by imaging tools in completely asymptomatic subjects or in subjects with nonspecific constitutional symptoms such as fever, asthenia, anorexia, and/or weight loss [13, 15, 35].

### 4.4. ECG Changes and Rhythm Disorders

Transitory changes in ventricular repolarization in 12-lead ECGs following extracardiac impingement are often reported in the literature. Although the exact mechanisms of such ECG findings are not yet completely understood, a number of plausible hypotheses have been put forward including local myocardial inflammation, pressure overload, microvascular disruption, transient vasospasm, and epicardial extrinsic compression [57–59]. Arvind and colleagues reported the case of a patient with chest pain and a large HH compressing the LA with ECG detection of ST-segment depression in lateral leads, which gradually regressed after nasogastric drainage and surgical repair [60]. Harada et al. described the case of a patient with imaging detection of both LA and LV compression by an enlarging HH with transient ST-segment elevation and hyperacute T-wave in anterior leads, which gradually disappeared without any treatment [40]. In the same way, Tyagi and coworkers reported the case of a young man with an esophageal meat bolus compressing the LA as well as ECG detection of ST-segment elevation in lateral leads and paroxysmal 2:1 atrioventricular block, which normalized after endoscopic treatment [34]. Furthermore, changes in physiological ECG morphology have also been reported in patients undergoing retrosternal esophageal reconstructive surgery. In this clinical scenario, the disruption of physiological anatomical landmarks between the heart and esophagus may lead to a broad spectrum of ECG manifestations [41, 46, 61]. Sasaki and Nakazato reported a case of iatrogenic Brugada-like ECG pattern detected after retrosternal reconstructive surgery for esophageal cancer, likely related to direct mechanical compression or postsurgical inflammation of the right ventricular outflow tract [62]. In a similar clinical setting, Kamimura and colleagues described respirophasic changes in ventricular repolarization in the 12-lead ECG of a patient with retrosternal esophageal reconstruction, caused by the pendulum-like cardiac motion to and from the anterior chest wall respectively according to the respiratory cycle [44]. Moreover, paroxysmal rhythm disorders, mainly related to transient supraventricular arrhythmias, have also occasionally been reported in this patient population [10, 19, 63]. The close proximity of gastroesophageal structures to the LA makes it prone to direct mechanical irritation, neural derangement, and inflammation, increasing the risk for atrial tachyarrhythmias. These findings were consistent with previous reports; a retrospective analysis by Roy et al. showed an epidemiologic interplay between HH and the development of atrial fibrillation in subjects aged < 55 years due to cardiac compression and the irritative arrhythmogenic trigger [64]. In the same way, Schilling and Kaye reported the suppression of paroxysmal atrial flutter by repairing a large paraesophageal HH [65]. Finally, the close anatomical relationship between the heart and GI structures may also be responsible for triggering ventricular arrhythmias. Gnanenthiran and colleagues reported a case of HH-induced ventricular tachycardia triggered by distortion of the mitral annular ring and aortomitral continuity, adjacent to the basal portion of the LV [66]. Similarly, Akdemir et al. described the case of a patient with syncopal-induced ventricular tachycardia during the postprandial period and imaging detection of extrinsic LV compression by a large HH, with improvement of symptoms after HH surgery [67]. In such clinical scenarios, several mechanisms play a relevant role in arrhythmogenesis. Cardiac distortion and myocardial irritation by extrinsic mechanical impingement, along with the neural impact mediated by the autonomic nervous system, provide a proarrhythmic substrate for inducing abnormal automatism and triggered activity. Other hypotheses include the development of an area of relative ischemia and conduction block, following persistent cardiac compression, causing reentry [66, 68].

### 4.5. Imaging Tools

**Figure 7:**
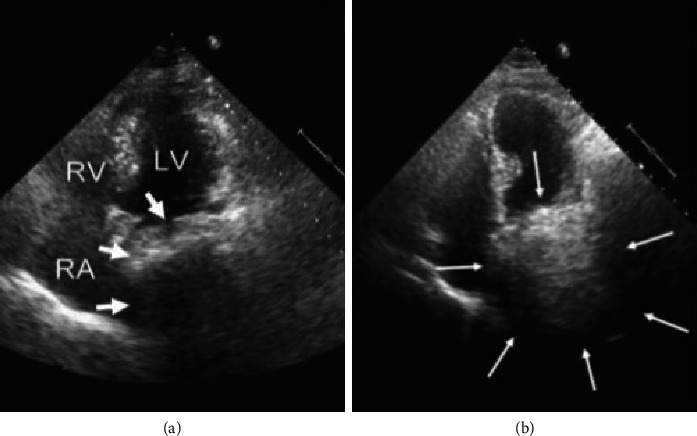
(a) Transthoracic echocardiography, apical 4-chamber view showing a mass of uncertain origin (intra-atrial vs. extracardiac) at left atrial posterior wall level (large arrows). (b) The same echocardiographic projection immediately after ingestion of a carbonated beverage showing the mass entirely filled with bubbles, thus confirming its extracardiac location (small arrows) (adapted from Bouzas-Mosquera et al.) [74]. Abbreviations: LV: left ventricle; RA: right atrium; RV: right ventricle.

**Figure 8:**
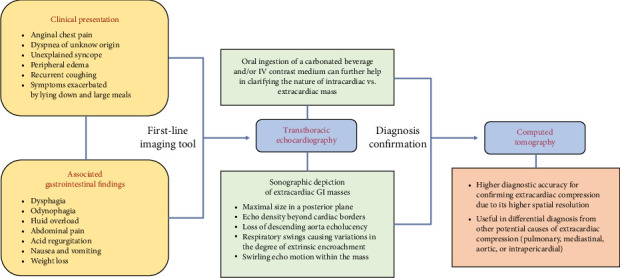
Flowchart summing up a proposed decision-making approach in this patient population.

A comprehensive diagnostic imaging approach plays a pivotal role in this clinical context, in order to ensure timely diagnosis, rapidly determine a decision-making strategy, and avoid inappropriate treatments. To date, CT is considered the best available tool for confirming the diagnosis of extracardiac compression by adjacent anatomical structures, due to its higher spatial resolution compared to other imaging tools. Additionally, it provides differential diagnosis from other etiologies, including pulmonary, mediastinal, aortic, or intrapericardial anatomical structures. Transthoracic echocardiography is considered the preferred first-line imaging tool as it is widely available, noninvasive, and cost-effective and provides useful insights into a large percentage of subjects with GI symptoms and a suspected clinical presentation, although a second-line imaging tool is generally required for confirming diagnosis [69]. Gastroesophageal lesions can hinder sonographic detection of the cardiac anatomy and simulate the appearance of intracardiac masses—e.g., thrombi, tumors, infective vegetations, embryonic residues, or artifacts—or other posterior mediastinal structures including mediastinal tumors or inflammations, vascular abnormalities, pleural or pulmonary diseases, and intrapericardial lesions [2, 70, 71]. Several echocardiographic features may help in distinguishing between intra-atrial masses and extrinsic gastroesophageal structures: (i) detection of the maximal size of the mass in a posterior imaging plane; (ii) the appearance of the echo density of the mass beyond the borders of the atria with angulation of the transducer, as it is a posterior mediastinal structure, separate from the heart; (iii) loss of the normally sharply defined echolucency of the descending thoracic aorta, posterolateral to the LA or posterior to the atrioventricular groove in the apical 4-chamber and long-axis views, due to the superimposition of a large ill-defined extrinsic echogenicity; (iv) respiratory swings, causing variations in the degree of encroachment of the extrinsic mass on the posterior aspect of the LA, because of the cyclic widening and narrowing of the atrial space between the aortic root and the anterior border of the digestive structure during respiratory fluctuations; (v) the “swirling” echo motion within a mass characterized by a continuous change in echo reflections from the contents of the mass exacerbated by the consumption of a carbonated beverage, thus resulting in a swirling effect of fluid and food particles interspersed with air within the mass [47, 72, 73]. Oral ingestion of a carbonated beverage can further help in clarifying the nature of a mass abutting the heart [74] (Figure 7). Additionally, intravenous infusion of an echocardiographic contrast medium may facilitate the characterization of the investigated structure provided the degree of enhancement is comparable to the vascular density. This is also the most reliable solution in cases of esophageal motor disorders, like achalasia, where dysphagia causes the retention of food and liquids in the esophagus and often limits the transit of ingested fluids [75]. Transesophageal echocardiography has been occasionally reported to aid in confirming the diagnosis of extracardiac compression, particularly when the extrinsic mass has a thick inner lining resembling the gastric mucosa. However, this methodology is not always successful [76, 77]. Despite the excellent contrast resolution and tissue characterization of magnetic resonance imaging, its use in defining extrinsic cardiac impingements by adjacent anatomical structures is still limited, because of its lower spatial resolution, compared to CT [78, 79]. A proposed decision-making approach for this patient population is provided on the basis of suspecting clinical findings and available imaging tools, in order to provide timely diagnosis and better categorize this kind of patient (Figure 8).

## 5. Conclusions

Extrinsic heart compression by GI structures encompasses a broad spectrum of clinical manifestations, depending on the underlying etiologies and cardiac chambers involved. Transitory electrocardiographic changes and heart rhythm disturbances may also be found in this kind of patient. Diagnostic workup often requires a comprehensive imaging approach, in order to avoid misleading interpretations and inform appropriate decision-making strategies.

## Figures and Tables

**Table 1:** Characterizing features of GI findings causing extracardiac compression.

	Characterizing features
Hiatal hernia	Most common gastrointestinal cause of extracardiac compression
	Main pathogenic mechanisms include the following:
	(i) Gastroesophageal reflux disease (causing traction of the gastroesophageal junction and gastric herniation)
	(ii) Chronic positive pressure of the esophageal diaphragmatic hiatus (which predisposes to gastric displacement into the chest)

Achalasia	Esophageal motor disorder in which impaired relaxation of the low esophageal sphincter due to inhibitory neuron degeneration leads to an increased basal sphincter pressure and distension of the distal esophagus, with no visceral displacement

Esophageal tumors	Primitive or secondary esophageal neoplasms inducing extracardiac compression due to their extrinsic mass effect

Iatrogenic compressions	A wide range of interventional procedures may potentially be responsible for extracardiac compression, including laparoscopic adjustable gastric banding, esophageal reconstructive surgery, and stent placement either as a palliative treatment or for the treatment of nonneoplastic lesions

Diaphragmatic herniations	Congenital (prevalent in childhood)
	(i) Bochdalek hernia (> 90%) posterolateral left-side location; generally symptomatic
	(ii) Morgagni–Larrey hernia (< 5%) anterior right-side location; often asymptomatic
	Traumatic (following blunt traumas or penetrating injuries)

Esophageal bolus	Favored by impaired esophageal peristalsis (due to either structural diseases or neuromuscular disorders)

Esophageal hematoma	Liable conditions include a sudden increase in esophageal pressure (e.g., during swallowing or vomiting), endoscopic procedures, bleeding disorders, or predisposing therapies

Esophageal angioedema	Causing a sudden accumulation of interstitial fluid and vasodilatation of the mucus membrane in the esophagus, resulting in esophageal swelling and extrinsic mass effect

## Data Availability

The data supporting the findings of this review are available from the corresponding author upon reasonable request.
